# Microfluidic hydrodynamic focusing synthesis of polymer-lipid nanoparticles for siRNA delivery

**DOI:** 10.18632/oncotarget.18281

**Published:** 2017-05-30

**Authors:** Xueqin Huang, Robert J. Lee, Yuhang Qi, Yujing Li, Jiahui Lu, Qingfan Meng, Lesheng Teng, Jing Xie

**Affiliations:** ^1^ School of Life Sciences, Jilin University, Changchun, Jilin 130023, China; ^2^ Department of Chemistry and Pharmacy, Zhuhai College of Jilin University, Zhuhai, Guangdong, 519041, China; ^3^ Division of Pharmaceutics, College of Pharmacy, The Ohio State University, Columbus, Ohio 43210, USA

**Keywords:** microfluidic, polymer, lipid nanoparticles, siRNA delivery, cancer treatment

## Abstract

Small interfering RNAs (siRNAs) are promising as therapeutics for intractable diseases such as cancer. However, efficient and safe delivery of siRNAs *in vivo* remains a challenge. Polymer-lipid hybrid nanoparticles (P/LNPs) have been evaluated for therapeutic delivery of siRNA. In this study, a microfluidic hydrodynamic focusing (MF) system was used to prepare P/LNPs loaded with VEGF siRNA. P/LNPs made by MF were smaller in particle size and had narrower size distribution compared to P/LNPs formed by bulk mixing (BM). MF-synthesized P/LNPs demonstrated low vehicle cytotoxicity and potent tumor cell inhibition *in vitro*. In addition, P/LNPs produced by the microfluidic chip exhibited prolonged blood circulation and increased AUC after i.v. injection compared to free siRNA. Furthermore, P/LNPs synthesized by MF induced greater down-regulation of VEGF mRNA and protein levels as well as greater tumor inhibition in a xenograft tumor model. Taken together, P/LNPs prepared by MF have been shown to be an effective and safe therapeutic siRNA delivery system for cancer treatment both *in vitro* and *in vivo*.

## INTRODUCTION

VEGF overexpression has been shown to increase angiogenesis, which promotes proliferation and metastasis of cancer cells [1, 2]. Therefore, down regulation of VEGF using siRNA is a promising therapeutic strategy for cancer. However, *in vivo* delivery of siRNA is challenging. [3] Because of their relative safety, many non-viral delivery systems have been evaluated for siRNA delivery [4–9]. Both polycation polyethylenimine (PEI) and cationic lipids have been used for siRNA delivery. They form electrostatic nanocomplexes with siRNA [10, 11]. However, the nanocomplexes have limited stability *in vivo* and can be cleared from circulation by the reticuloendothelial system (RES) [12]. Polyethylene glycol (PEG) coating has been shown to reduce the particle size and improve the stability of these nanocomplexes [13, 14]. Further improvements in siRNA delivery efficiency may be possible if the process of nanocomplex synthesis can be optimized.

Conventional methods for nanocomplex synthesis is based on bulk-mixing (BM) of cationic components and siRNA solutions [15–19]. It is an uncontrolled process usually involving multiple steps, therefore, has poor reproducibility. In the last decade, microfluidic hydrodynamic focusing (MF) devices have been introduced to nanoparticle synthesis [17, 20–22]. MF has been shown to produce nanoparticles that are smaller in size and narrower in size distribution.

Low molecular weight PEI has been shown to be effective for siRNA delivery and to have low cytotoxicity [23, 24]. In this study, PEI-800 was combined with cationic lipids to compose polymer-lipid hybrid nanoparticles (P/LNPs), with the composition of DODMA/egg PC/Chol/DSPE-PEG2000/PEI-800 at a molar ratio of 40/19/35/1/5 [25]. Incorporation of DSPE-PEG2000 was aimed at increasing the circulation time of P/LNPs when used *in vivo*. Moreover, P/LNPs were prepared by MF with sonication using a flow rate of 0.8 mL/min. These conditions have been previous identified as optimal parameters for generating P/LNPs with optimal characteristics. Physicochemical properties and cytotoxicity of siRNA-loaded P/LNPs were investigated. *In vivo* pharmacokinetics and pharmacodynamics of P/LNPs were evaluated in tumor-bearing mice. Gene silencing activity of P/LNPs was evaluated. The data showed that P/LNPs containing VEGF siRNA produced by the MF method (P/LNPs-siRNA-MF) induced greater cytotoxicity *in vitro* and greater tumor inhibition *in vivo* compared to those made by BM method (P/LNPs-siRNA-BM).

## RESULTS

### Characterization of P/LNPs

Mean particle size, polydispersity index (PDI) and zeta potential of the various P/LNPs were determined by dynamic light scattering (DLS) and zeta potential measurement. In this study, we performed the synthesis of P/LNPs at a V_f_ of 0.8 ml/min in the presence of sonication. The results obtained are shown in Table 1. LNPs-siRNA-BM (without PEI-800) had a size of 177.5 nm and P/LNPs-siRNA-BM (with PEI-800) had a size of 194 nm. It appears that incorporation of PEI-800 into LNPs slightly increased the particle size as well as the PDI. The particle size of the P/LNPs-siRNA-MF (with PEI-800) was 106.4 nm with a PDI of 0.126, indicating a narrower size distribution. The zeta potential values of P/LNPs-siRNA-MF were slightly lower than those of LNPs-siRNA-BM. Therefore, the MF method produced LNPs with a narrower particle distribution and lower surface charge.

**Table 1:** Properties of nanoparticles prepared by MF and BM methods

Samples	Mean Diameter (nm)	PDI	Zeta potential (mV)
LNPs-siRNA-BM P/LNPs-siRNA-BM	178 ± 17 194 ± 23	0.217 0.232	3.9 ± 0.2 18.7 ± 3.6
P/LNPs-siRNA -MF	106 ± 8	0.126	10.4 ± 0.4

Samples were studied by DLS and zeta potential measurement. Each run was done in triplicate and data are expressed as mean ± SD.

Abbreviations: LNPs-siRNA-BM, siRNA-loaded LNPs (without PEI-800) formed by bulk mixing method; P/LNPs-siRNA-BM, siRNA-loaded P/LNPs (with PEI-800) formed by bulk mixing method; P/LNPs-siRNA-MF, siRNA-loaded P/LNPs (with PEI-800) formed by microfluidic method; PDI, Polydispersity index.

### P/LNP vehicle cytotoxicity assay

Cytotoxicity of blank P/LNPs was tested in HepG-2 cells using an MTT assay. Cell viability at 12h, 24 h, 48 h, and 72 h after adding P/LNPs was calculated and shown in Figure 2A. At the first time point, with blank P/LNPs (12 h), cell viability was nearly 100%. After 24 h incubation (20 µg/mL lipids), P/LNPs-MF killed only 8.6 ± 4.5% of cells. Meanwhile, the same quantity of P/LNPs-BM killed 10.4 ± 3.3% cells. At 48 h and 72 h, HepG-2 cells still showed high viability. The results revealed that blank P/LNPs, regardless of method of synthesis, were not cytotoxic.

**Figure 1:**
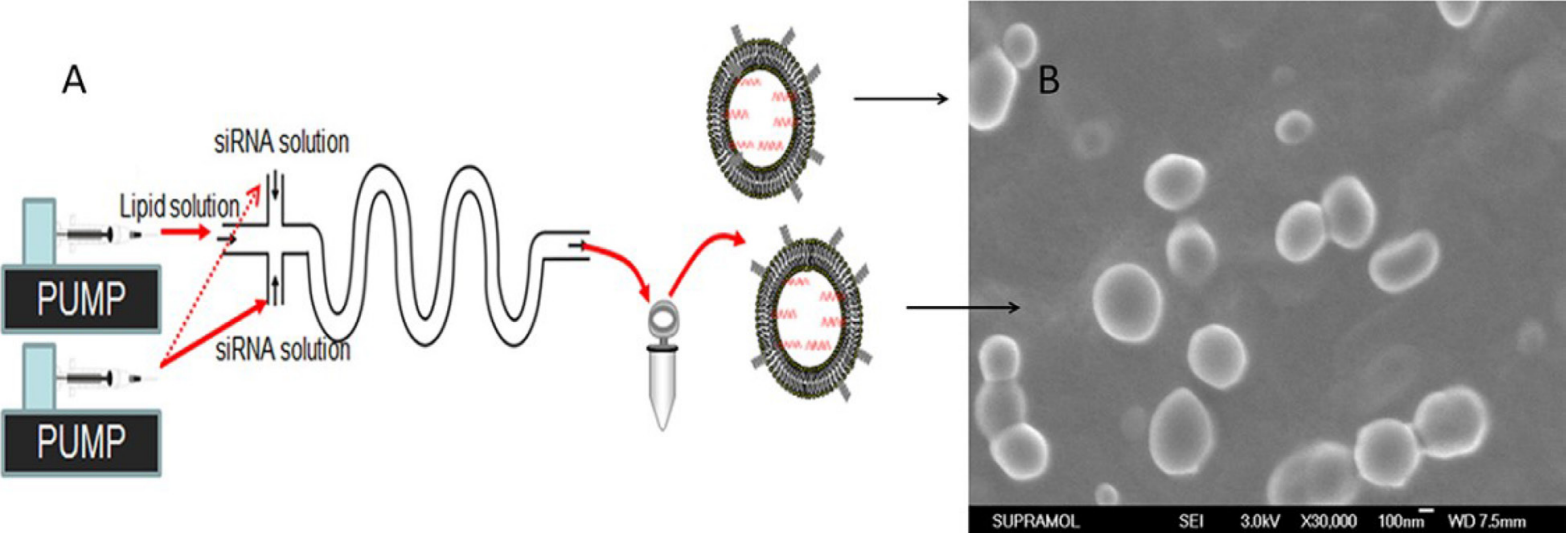
Schematic of P/LNPs synthesis using a microfluidic device The device consisted of three inlet ports and one outlet port and was control by two syringe pumps. siRNA solutions were introduced from inlets 1 and 3, and the lipid solution was injected from inlet 2 and was mixed with siRNA solution through an S-style MF channel at 0.8 mL/min flow rate. Resulting P/LNPs was collected at the outlet port, followed by sonication and dialysis.

**Figure 2:**
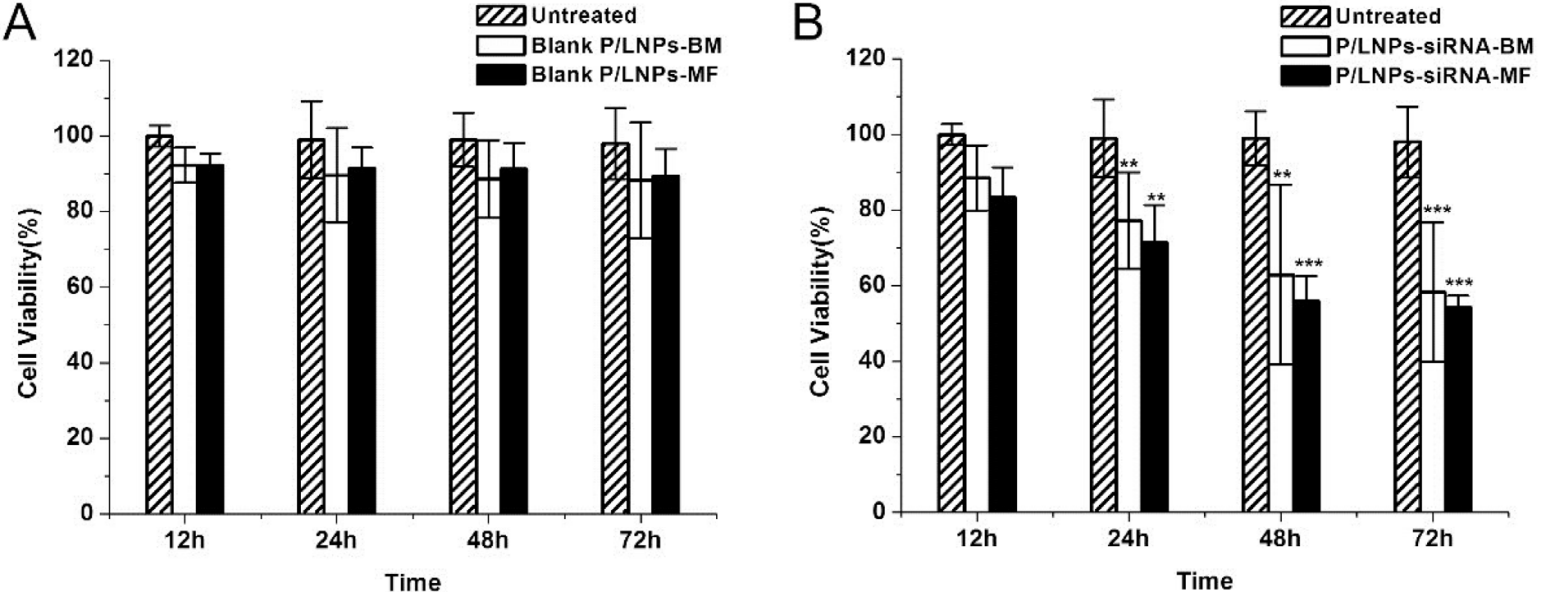
MTT assay of blank P/LNPs and siRNA-loaded P/LNPs on HepG-2 cells Cells were incubated with (**A**) blank P/LNPs or (**B**) siRNA-loaded P/LNPS for 12h, 24 h, 48 h or 72 h in an atmosphere containing 5% CO_2_ at37°C. All values were presented as mean ± SD (^*^ indicates *p* < 0.05, ^**^ indicates *p* < 0.01 and ^***^ indicates *p* < 0.001). Abbreviations: blank P/LNPs-BM, P/LNPs-BM without siRNA, blank P/LNPs-MF, P/LNPs-MF without siRNA, P/LNPs-siRNA-BM, siRNA-loaded P/LNPs-BM, P/LNPs-siRNA-MF, siRNA-loaded P/LNPs-MF.

### Growth inhibition of tumor cell

The effect of VEGF siRNA-loaded P/LNPs on growth of HepG-2 cells was investigated. The results revealed that the loss of the cell viability was closely correlated with incubation time of P/LNPs (Figure 2B). The growth of tumor cells was reduced even at 12h after adding P/LNPs-siRNA-MF. After 72 h incubation, P/LNPs-siRNA-MF treated cells showed significantly greater growth inhibition with only 54 ± 3.1% survival rate relative to the control. In addition, the cell viability of P/LNPs-siRNA-BM treatment group was higher than that of P/LNPs-siRNA-MF group. These results clearly showed that P/LNPs-siRNA-MF had significant cytotoxicity.

### Intracellular trafficking of P/LNPs

**Figure 3:**
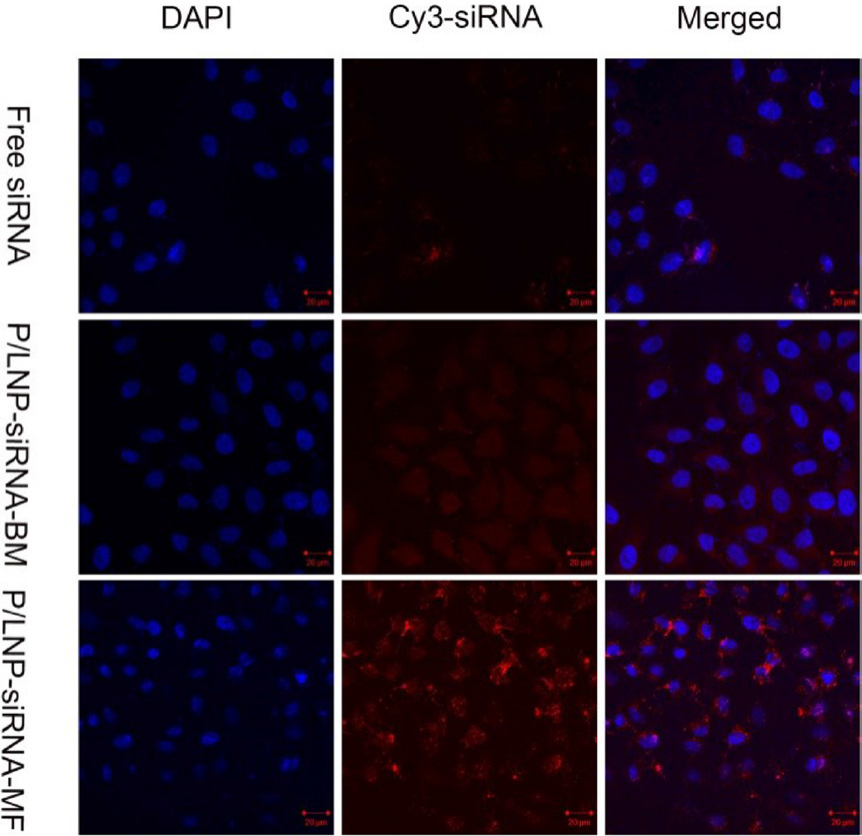
Intracellular trafficking of P/LNPs The analysis of P/LNPs mediated siRNA delivery in HepG-2 cells was performed by confocal microscopy. Cell nuclei were counterstained with DAPI. Abbreviations: P/LNPs-siRNA-MF, Cy3-siRNA-loaded P/LNPs-MF, P/LNPs-siRNA-BM, Cy3-siRNA-loaded P/LNPs-BM, Cy3-siRNA, Cy3-labeled siRNA.

To identify the cellular uptake of P/LNPs, the cells treated Cy3-labeled siRNA in P/LNPs were evaluated by confocal microscopy at 4 h after addition, as shown in Figure 3. Without any protection, few free siRNA were delivered into cytoplasm. In contrast, more siRNA (red fluorescence) was clearly visualized in cells treated with P/LNPs-siRNA-MF compared with treatment by P/LNPs-siRNA-BM, indicating more efficient uptake of P/LNPs-siRNA-MF.

### Pharmacokinetic studies

**Figure 4:**
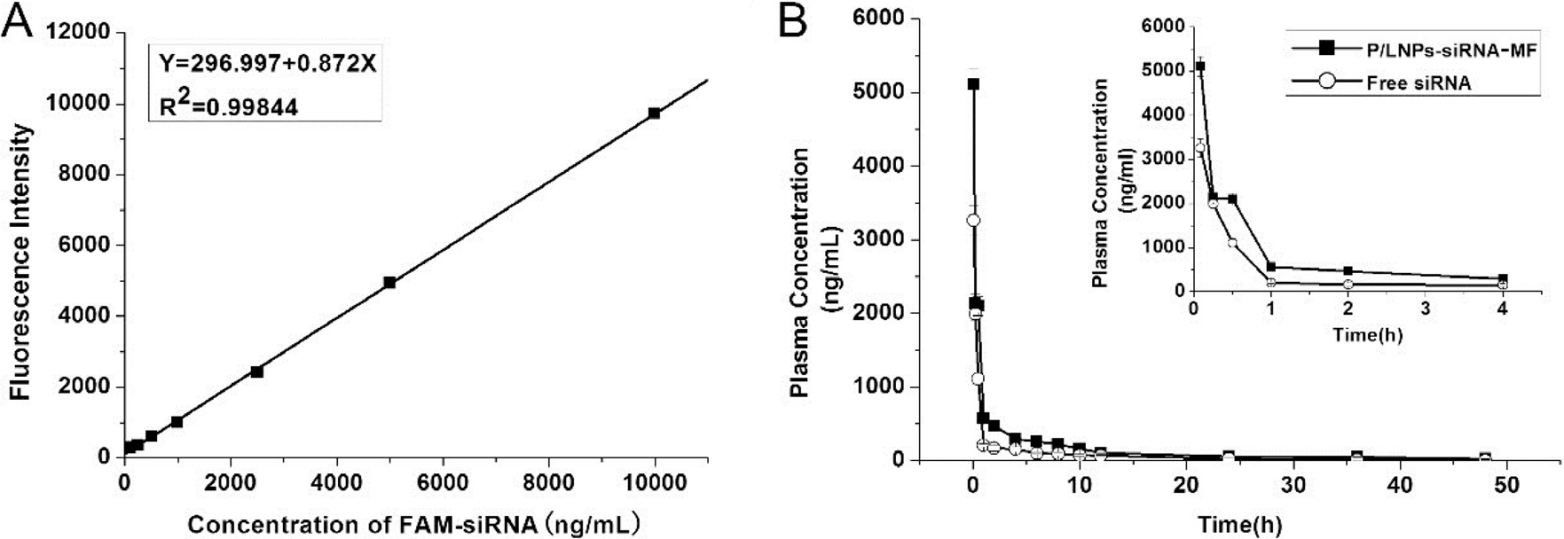
Pharmacokinetics of P/LNPs in Wistar rats (**A**) Standard curve of Fluorescence Intensity versus Concentration of FAM-siRNA. (**B**) Plasma concentrations of P/LNPs-siRNA-MF and free FAM-siRNA after i.v. administration. The data are presented as means ± SD (*n* = 3). Abbreviations: P/LNPs-siRNA-MF, FAM-siRNA-loaded P/LNPs-MF; FAM-siRNA, FAM-labeled siRNA.

Free siRNA is rapidly cleared due to presence of nuclease and excretion. P/LNPs covered with a protective PEG layer may produce not only longer circulation time, but also greater passive tumor accumulation *in vivo* as a result of enhanced permeability and retention. PK properties of P/LNPs, including maximum plasma concentration (C_max_), half-life (T_1/2_), area under the curve (AUC_0–∞_), total body clearance (CL), and mean residence time (MRT_0–∞_) were calculated and presented in Figure 4 and in Table 2. P/LNPs-siRNA-MF (FAM-siRNA-loaded P/LNPs-MF) was intravenously injected into Wistar rats. Free FAM-siRNA was used as a control. Figure 4 shows that C_max_ obtained with P/LNPs-siRNA-MF was considerably greater than that of free siRNA at the first time point. The plasma concentration of free siRNA group decreased rapidly subsequently. At 30 min, plasma concentration was approximately 1000 ng/mL, corresponding to less than half of the values observed following P/LNPs-siRNA-MF treatment (2000 ng/mL). This suggested that free siRNA was rapidly eliminated from the blood circulation *in vivo* would not be capable of exerting any therapeutic function. However, the plasma concentration of P/LNPs-siRNA-MF group declined sharply at first and then the rate of decrease slowed down. Table 2 also showed that P/LNPs-siRNA-MF was cleared slowly from the circulation compared to free FAM-siRNA, resulting in longer t_1/2_ values. AUC_0–∞_ and MRT_0–∞_ values of P/LNPs-siRNA-MF were significantly higher than those of free FAM-siRNA. Among all formulations, P/LNPs-siRNA-MF presented the longest MRT, the largest AUC and the lowest CL, indicating a long circulation time.

**Table 2:** Plasma pharmacokinetic parameters

	C_max_ (ng/mL)	T_1/2_ (h)	Cl (mLkg1)	AUC_0–∞_	MRT_0–∞_
Free FAM-siRNA	3264	12.8	46.6	5543	18.1
P/LNPs-siRNA-MF	7107	28.9	13.1	14345	28.6

Pharmacokinetic parameters of P/LNPs-siRNA-MF and free FAM-siRNA were collected and calculated after i.v. administration.

Abbreviations: C_max_, maximum plasma concentration; T_1/2_, half-life; Cl, total body clearance; AUC_0–∞_, area under the curve; MRT_0–∞_, mean residence time; P/LNPs-siRNA-MF, FAM-labeled siRNA-loaded P/LNPs-MF, FAM-siRNA, FAM-labeled siRNA.

### Tumor growth suppression

**Figure 5:**
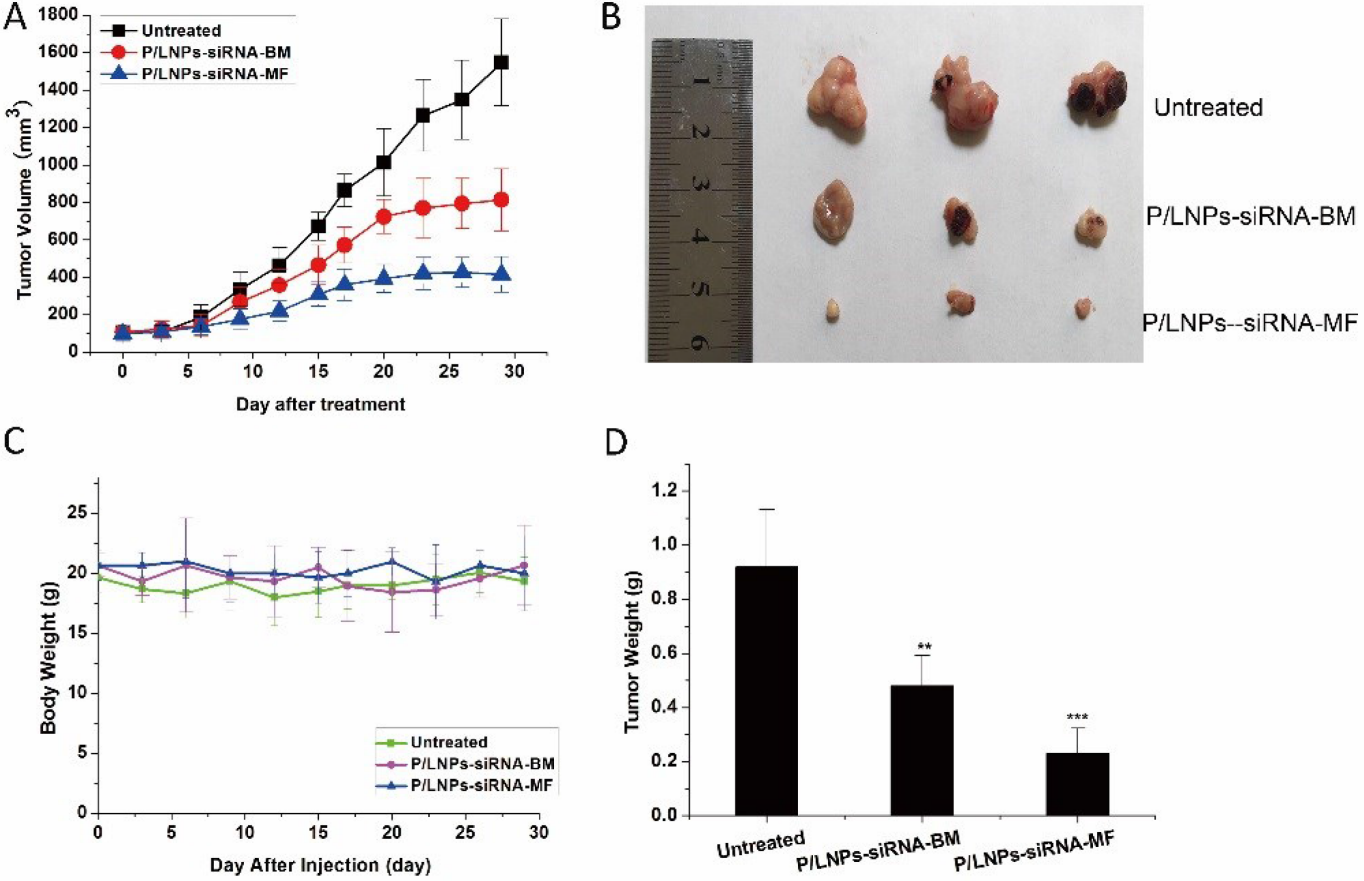
*In vivo* Suppression of tumor growth by various P/LNPs in HepG-2 tumor bearing BALB/C-nude mice (**A**) Tumor volumes were measured by caliper three times a week after P/LNPs-siRNA-BM, P/LNPs-siRNA-MF, or saline treatment. (**B**) Images of the harvested tumors at the time of sacrifice. (**C**) Body weights of mice were monitored during the treatment. (**D**) The average weight of tumors collected at the end of treatment. (^*^p < 0.05, ^**^p < 0.01, ^***^p < 0.001). Abbreviations: P/LNPs-siRNA-BM, siRNA-loaded P/LNPs-BM; P/LNPs-siRNA-MF, siRNA-loaded P/LNPs-MF.

To study the therapeutic efficacy of P/LNPs *in vivo*, a murine xenograft model was used. BALB/C-nude mice was subcutaneously inoculated with HepG-2 cells prior to experiment. Average tumor volume and body weight were monitored throughout the study. When the tumor has grown to around 100 mm^3^, P/LNPs were administered via the tail vein at a dosage of 2.5 mg/kg siRNA every 3 days. After the completion of the studies, tumors were dissected from all mice and weighed. The results showed that P/LNPs-siRNA-BM kept tumor growth to 600∼800 mm^3^, whereas tumor size remained at 100∼200 mm^3^ and decreased in size in later time points in mice treated with P/LNPs-siRNA-MF. These revealed that both P/LNPs-siRNA-MF and P/LNPs-siRNA-BM suppressed the growth of tumors, but at the same dose regimen, the growth rate of tumor in mice treated with P/LNPs-siRNA-MF was slower compared with those treated with P/LNPs-siRNA-BM (Figure 5A, 5B). No statistically significant difference in body weight was observed between P/LNPs and control groups during the treatment period (Figure 5C). At the end of treatment, tumor weights of P/LNPs-siRNA-MF and P/LNPs-siRNA-BM treated group were 0.23 ± 0.09 and 0.48 ± 0.11 g, respectively (Figure 5D). It seems that P/LNPs-siRNA-MF exhibited greater inhibition of tumor growth than P/LNPs-siRNA-BM.

### *In vivo* gene silencing and histopathology

**Figure 6:**
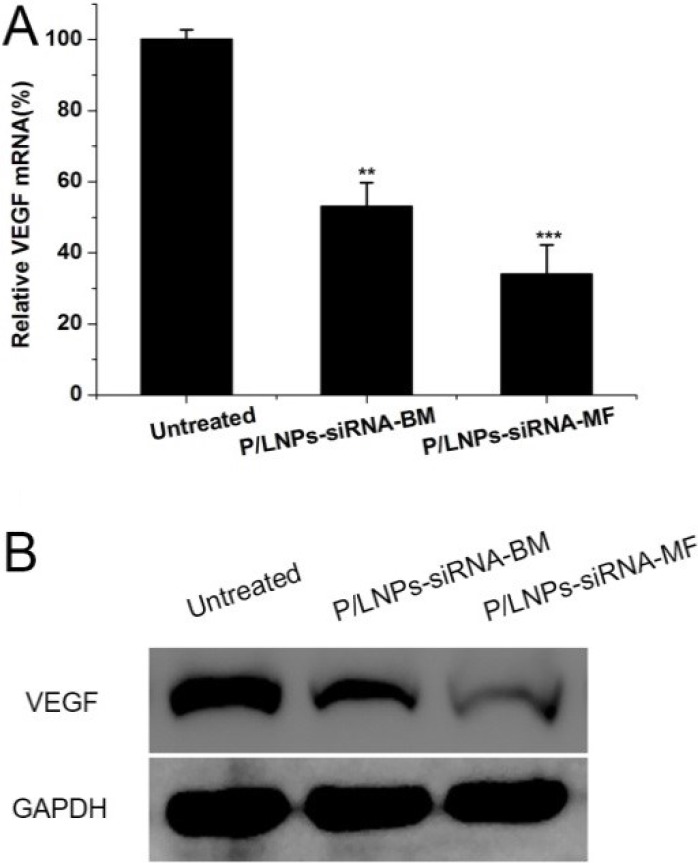
*In vivo* down-regulation of VEGF by siRNA in P/LNPs Notes: (**A**) The level of VEGF mRNA determined by qRT-PCR. (**B**) VEGF protein expression determined via Western blot analysis. Data represents mean ± SD (*n* = 3). ^*^ indicates *p* < 0.05, ^**^ indicates *p* < 0.01 and. ^***^ indicates *p* < 0.001. Abbreviations: P/LNPs-siRNA-BM, siRNA-loaded P/LNPs-BM; P/LNPs-siRNA-MF, siRNA-loaded P/LNPs-MF.

Based on previously shown gene silencing effects of P/LNPs *in vitro*, these effects were evaluated in tumor-bearing mice *in vivo*. As shown in Figure 6A, down regulation of VEGF in tumor by P/LNPs-siRNA-MF was 69%, while the value for P/LNPs-siRNA-BM was only 44%. Overall, the VEGF mRNA level in the P/LNPs-siRNA-BM treatment group was moderately lower than in the control group, whereas P/LNPs-siRNA-MF more effectively inhibited the VEGF mRNA expression. Western blot analysis also showed similar results on the VGRF protein level (Figure 6B). P/LNPs-siRNA-MF down-regulated VEGF more efficiently than P/LNPs-siRNA-BM, indicated that P/LNPs-siRNA-MF could deliver siRNA more efficiently into the tumor site.

**Figure 7:**
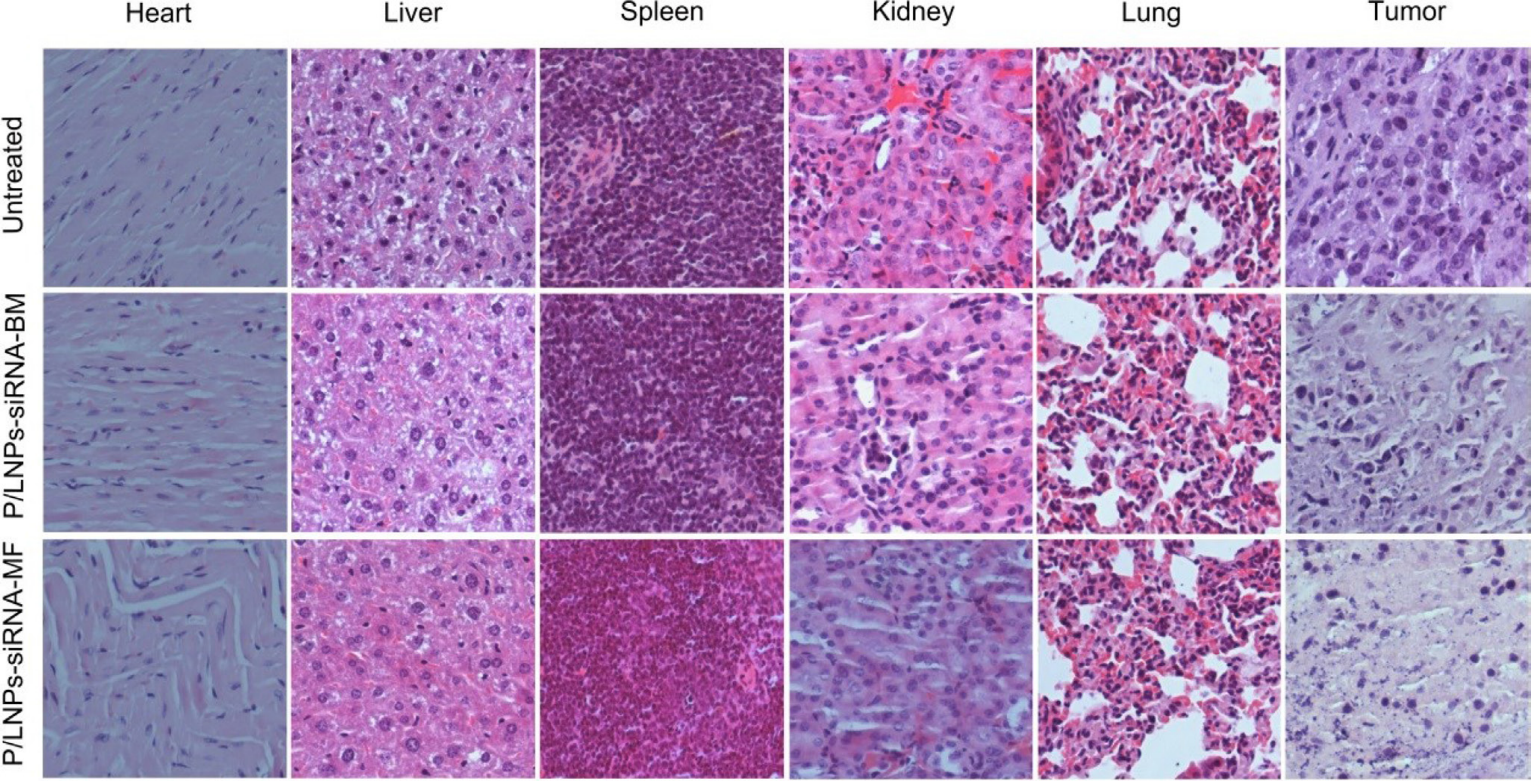
Histopathology of tissue sections stained with hematoxylin/eosin (H&E) Heart, Liver, Spleen, Lung, Kidney, Tumor tissue were collected and stained by hematoxylin/eosin (H&E) after the last treatment. Abbreviations: P/LNPs-siRNA-BM, siRNA-loaded P/LNPs-BM; P/LNPs-siRNA-MF, siRNA-loaded P/LNPs-MF.

Additionally, tumor sections were stained with H&E for further pathological analysis (Figure 7). In the control group, high density tumor cells with nuclei were detected clearly. In contrast, tumor cells appear apoptotic and less dense in the P/LNPs-siRNA-BM group, although many viable cells remained. Finally, extensive tumor cells appeared to be severely damaged with loss of nuclei in the P/LNPs-siRNA-MF treatment group. These results suggested that the P/LNPs-siRNA-MF induced much tumor cell death and reduced the density of tumor tissue, therefore, is effective for cancer therapy. Meanwhile, histopathology sections of the other tissues did not show any visible differences among three treatment groups.

### Systemic toxicity analysis

**Figure 8:**
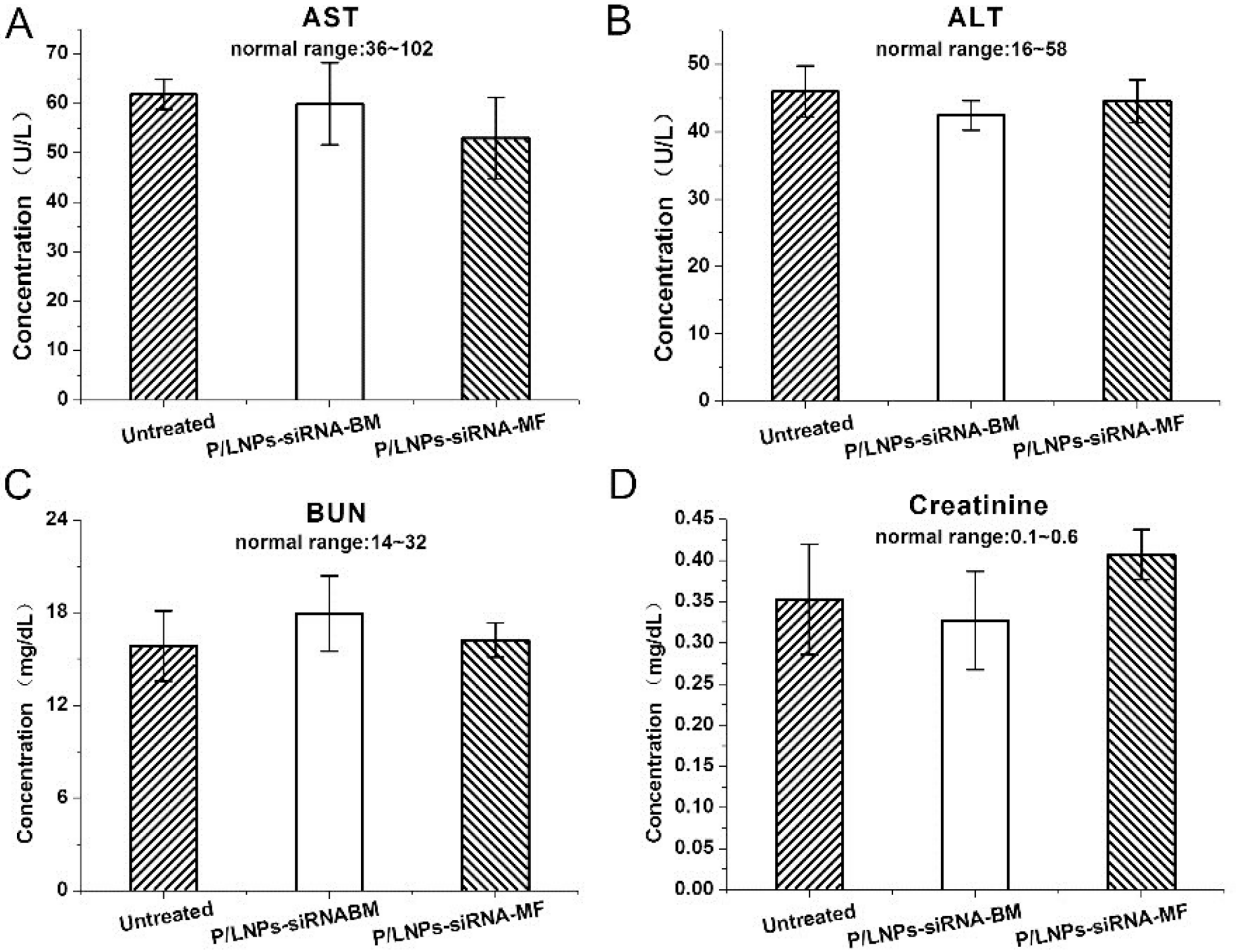
Blood biochemical parameters of mice treated P/LNPs *In vivo* toxicity assay by measuring the levels of (**A**) AST, (**B**) ALT, (**C**) BUN and (**D**) creatinine in serum of mice 48 h after intravenous injections of P/LNPs. Normal ranges of parameters are presented. Data are expressed as mean ± SD (*n* = 3). Abbreviation: AST, aspartate aminotransferase; ALT: alanine aminotransferase; BUN: blood urea nitrogen; P/LNPs-siRNA-BM, siRNA-loaded P/LNPs-BM; P/LNPs-siRNA-MF, siRNA-loaded P/LNPs-MF.

Serum analyses were used to assess any abnormality in liver and kidney function after P/LNPs treatment. As shown in Figure 8, normal serum alanine aminotransferase (ALT) and aspartate aminotransferase (AST) levels following treatment indicated that there was no significant liver damage caused by P/LNPs. In addition, serum blood urea nitrogen (BUN) and creatinine levels for mice treated by P/LNPs were similar to those of the control, suggesting no abnormal changes in kidney functions after P/LNPs injection.

## DISCUSSION

Over-expression of VEGF promotes tumor angiogenesis [26, 27]. Therefore, targeting VEGF using siRNA is a promising therapeutic strategy. In our previous report, P/LNPs of the same composition as used in this study were shown to achieve high gene silencing efficiency *in vitro* [25]. In this report, to further evaluate the therapeutic effect of P/LNPs, *in vivo* studies were conducted.

MF is a relatively novel technique in nanoparticle synthesis [28–32]. Due to its ability to precisely manipulate and control LNP properties, we carried out P/LNPs synthesis using a MF system. Particle size is important because small size can facilitate tumor through extravasation and the enhanced permeation and retention (EPR) effect [33]. P/LNPs produced by MF method and by BM method with the size range of 110 to 200 nm enable them to extravasate and be internalized into the cell via endocytosis. Moreover, P/LNPs made by MF had a narrow particle size distribution, smaller particle size and lower surface charge compared to those made by BM, indicating that MF method could produce P/LNPs with superior physicochemical properties [34]. For *in vitro* experiment, P/LNPs-siRNA-MF were shown to deliver siRNA into the cytoplasm and cause tumor cell death as well as induce down-regulation of VEGF expression (data not shown).

Therefore, based on the promising *in vitro* data, we continued our study P/LNPs *in vivo*. It is known that If P/LNPs are quickly eliminated in serum, accumulation of siRNA at tumor site would be diminished. Thus, delivery vehicles with extended blood circulation lifetime and serum stability are indispensable to promote delivery at tumor sites and protect the siRNA molecule in serum against nucleases and excretion. PEGylation is capable of effectively diminishing clearance and prolonging circulation time, resulting in enhanced AUC, MRT and T_1/2_ of P/LNPs-siRNA-MF compared to free siRNA. Furthermore, we anticipate that increased blood circulation of P/LNPs *in vivo* will have a positive impact on its tumor suppression efficacy. This hypothesis was proven by comparing the antitumor activity of P/LNPs *in vivo*. As shown in Figure 4, exponential increase in tumor size was seen in the control group, with the tumor size reaching ∼1500 mm^3^. As expected, the P/LNPs-siRNA-MF treated group exhibited favorable tumor suppression compared to the P/LNPs-siRNA-BM treated group (31.64 versus 70.67 mm^3^/day), suggesting that P/LNPs-siRNA-MF was more efficacious.

P/LNPs already have been demonstrated to have gene silencing activity in HepG-2 liver cancer cells during *in vitro* studies. Here, we performed qRT-PCR and western blot analysis for silencing efficiencies at VEGF mRNA and protein levels in tumors in mice treated with P/LNPs *in vivo*. Consistent with the *in vitro* results, P/LNPs-siRNA-MF facilitated the greatest down regulation in gene expression at the mRNA and protein levels among the three groups. In addition, pathological analysis clearly showed that tumor tissue suffer severe damage after P/LNPs-siRNA-MF therapy, while no histological changes were detected in normal organ tissues indicating P/LNPs were not toxic. Taken together, it was confirmed that P/LNPs-siRNA-MF was the most effective approach for VEGF gene silencing as well as tumor suppression *in vivo*. The superior anti-tumor activity of P/LNPs-siRNA-MF *in vivo* likely could be attributed to nanoparticle synthesis using MF method. MF provides better mixing between P/LNPs and siRNA, resulting in uniform nanostructure and reduced surface charge of P/LNPs-siRNA-MF. With smaller particle size and narrower size distribution, P/LNPs-siRNA-MF was easier to accumulate at tumor site with leakier blood vessel than P/LNPs-siRNA-BM. On the other hand, low surface charge of P/LNPs-siRNA-MF is associated with improved P/LNP stability *in vivo* and reduced adsorption of serum proteins.

We further investigated whether P/LNPs induced toxicity *in vitro* and *in vivo*. MTT results show that no significant cytotoxicity was observed with blank P/LNPs *in vitro*. Meanwhile, serum analysis showed no significant systemic toxicity in mice treated with the P/LNPs-siRNA-MF or P/LNPs-siRNA-BM (Figure 7). These data showed that P/LNPs have low toxicity and has an excellent safety profile.

## MATERIALS AND METHODS

### Materials

1,2-Dioleyloxy-N,N-dimethyl-3-aminopropane (DODMA) was obtained from Corden Pharma (Cambridge, MA, USA). Egg L-α-phosphatidylcholine (egg PC), cholesterol (Chol) and 1,2-distearoyl-sn-glycero3-phosphoethanolamine -N-[methoxy(polyethylene glycol)-2000](DSPE-PEG2000) were purchased from Avanti Polar Lipids (Alabaster, AL, USA). Branched PEI with molecular weight of 800Da (PEI-800), 3-(4, 5-dimethylthiazol-2yl)-2,5-diphenyltetrazolium bromide (MTT), and cell culture media and supplies were purchased from Sigma-Aldrich (St. Louis, MO, USA). VEGF siRNA (sense sequence: 5′-GGAGUACCCUGAUGAGAUCdTdT-3′; antisense sequence: 5′-GAUCUCAUCAGGGUACUCCdTdT-3′) and 5′-FAM-siRNA were provided by Guangzhou RiboBio Co., Ltd. (Guangzhou, China).

### Tissue culture

HepG-2 cells, obtained from the American Type Culture Collection (ATCC) (Manassas, VA, USA), were cultured in the DMEM medium supplemented with 10% fetal bovine serum, 1% antibiotics and 1% antimycotics, at 37°C in a humidified atmosphere containing 5% CO_2_.

### Laboratory animals

Male nude mice (4 weeks old) and Wistar rats were purchased from the Laboratory Animal Center of Jilin University (Changchun, China) and housed in a room with controlled temperature and humidity. Standard chow and water were supplied to the animals under a 12 h light/dark cycle. All protocols used were approved by the Animal Care and Research Committee at Jilin University.

### Preparation of P/LNPs

P/LNPs were prepared both by an MF method and a BM method, as described previously. [35] Briefly, DODMA/egg PC/Chol/DSPE-PEG2000/PEI-800 at 40/19/35/1/5 (molar ratio) were dissolved in absolute ethanol to form a lipid/polymer solution. VEGF siRNA was dissolved in HEPES (20 mM HEPES, pH = 4). The weight ratio of lipids-to-siRNA used was 10:1. For nanoparticle synthesis by the BM method, the lipid/polymer solution was added into siRNA solution with stirring at 4°C. After sonication, ethanol was removed by dialysis using a MWCO 10 kDa Float-A-Lyzer against 20 mM HEPES (pH = 7.4) buffer for 2 hours.

For nanoparticle synthesis using the MF method, two syringe pumps and a 3-inlet microfluidic chip were used, as shown in Figure 1. siRNA solution was introduced at inlets 1 and 3. Meanwhile, lipid/polymer solution was injected through inlets 2, and was hydrodynamically focused by siRNA streams at the first fluid channel intersection. The flow rate of the streams was 0.8 mL/min. The resulting P/LNPs were collected at the outlet port, followed by sonication and dialysis as described above. Next, the P/LNPs-siRNA-MF and P/LNPs-siRNA-BM were sterilized by passing through a 0.22 μm pore-size syringe filter (Millipore, Billerica, MA, USA).

### Zeta potential and particle size measurements

Particle size, polydispersity index (PdI) and zeta potential of P/LNPs were analyzed on a NICOMP 380 ZLS analyzer from Particle Sizing Systems (Santa Barbara, CA, USA).

### Cytotoxicity assay

An MTT assay was used to evaluate cytotoxicity of nanoparticles *in vitro*. Cells were seeded at 6000 cells/well and cultured under standard conditions (37°C, 5% CO_2_). Then, the cells were treated with culture medium containing blank P/LNPs or P/LNPs loaded with siRNA for 12h, 24 h, 48 h, or 72 h. After incubation, medium was removed from the cells and 20 μL MTT reagent was added into each well and the plate was incubated for another 4 h. Then, the medium was replaced with 150 μL DMSO to dissolve blue formazan crystals formed and absorbance value was determined at 490 nm on a BioTek Synergy 4 Hybrid Microplate Reader. Cell viability (%) was calculated using the following equation, where A_sample_ and A_control_ represented absorbances of cells treated with a nanoparticle sample and blank medium, relatively [36].

$$\mathrm{Relative}\;\mathrm{cell}\;\mathrm{viability}\left(\%\right)=\frac{A_{sample}}{A_{control}}\times 100\%$$

### Confocal microscopy

To visualize the uptake of P/LNPs by tumor cells, confocal laser scanning microscopy was used. HepG-2 cells were seeded (3 × 10^4^cells/well), cultured for 24h and treated with Cy3-labeled siRNA (Cy3-siRNA) or P/LNPs-siRNA (Cy3-siRNA-loaded P/LNPs). After 4 h incubation, the cells were washed with PBS for three times and then fixed with 4% paraformaldehyde solution for 15 min. Then DAPI was added for the staining of nuclei. Finally, the cells were observed on a Zeiss710 LSMNLO Confocal Microscope from Carl Zeiss (Oberkochen, Germany [37]).

### Pharmacokinetic studies

Plasma pharmacokinetic analysis was performed in healthy male Wistar rats. Plasma concentrations were determined by measuring the fluorescence intensity of FAM-labeled siRNA (FAM-siRNA) in serum. Briefly, Rats were randomly divided into 2 groups of 5 mice per group and respectively injected with free FAM-siRNA and P/LNPs-siRNA-MF (FAM-siRNA-loaded in P/LNPs-MF) at a dose of 10 mg/kg. Then, 200 μL of blood samples from the ophthalmic vein were collected at 5 min, 15 min, 30 min, 1 h, 2 h, 4 h, 10 h, 24 h and 48 h after injection. Blood samples were stored at 4°C for 5min and then centrifuged at 10, 000rpm for 10min. Plasma supernatant was mixed with 1% SDS and heated to 95°C, followed by centrifugation at 10, 000rpm for 10 min. Fluorescence of supernatant was measured on a microplate reader (λ_ex_: 485 nm, λ_em_: 520 nm). Pharmacokinetics parameters including maximum plasma concentration (C_max_), half-life (t_1/2_), area under the curve (AUC_0-∞_), mean residence time (MRT_0-∞_) and total body clearance (CL) were calculated by non-compartmental analysis using WinNonlin version 5.2.1 software (Pharsight Corp.).

### Antitumor efficacy *in vivo*

A murine xenograft model was used to assess antitumor activity. Male nude mice were inoculated with HepG-2 cells at 6 × 10^6^ per animal subcutaneously in the right flank to establish tumors. Two weeks later, mice were randomly divided into 3 groups with 5 mice per group. Body weight and tumor size were measured every 3 days. When the average tumor volume reached ∼ 100 mm^3^, mice were injected with saline (control) or P/LNPs containing siRNA at a dose of 2.5 mg/kg via tail vein twice a week. Tumor volume (V) was calculated according to the following equation after measuring the width and length of each tumor using a caliper.

$$\mathrm{Tumor}\;\mathrm{volume}\left({\mathrm{mm}}^{3}\right)=\frac{\mathrm{Width}\;\times \;\mathrm{Length}}{2}$$

After the final treatment, the mice were sacrificed and tumors were harvested, weighed, imaged and analyzed for VEGF gene expression and histology.

### Analysis of gene expression

Tumors harvested were homogenized in a lysis buffer. Total protein and mRNA extracts were obtained using RIPA (Sigma-Aldrich) and RNAiso Plus (Takara) according to manufacturer’s protocols, respectively. qRT-PCR was used to measure mRNA levels. First, extracted total RNA was reverse-transcribed into cDNA. Then, the cDNA was subjected to qRT-PCR (CFX96T Real-time System, Bio-Rad) and amplified according to following conditions: 40 cycles including denaturation at 94°C for 1 min; annealing: 60°C for 1 min [38]; extension: 72°C for 1 min. For Western blot assay, 20 μg of total protein was subjected to electrophoresis on a sodium dodecyl sulfate polyacrylamide gel (90V for spacer gel, 120V for separation gel) and then transferred to a polyvinylidene fluoride (PVDF) membrane. The blotted membranes were incubated with specific primary antibodies overnight, and subsequently, incubated with secondary horseradish peroxidase (HRP)-labeled antibody for 4 h. After washing for 3 times, immunostaining was performed using an enhanced chemiluminescence (ECL) kit (GE Healthcare, UK) and then imaged on a Biospectrum600 Imaging System (Upland, CA, USA [39]).

### Histopathology staining

At 48 h after the final treatment, the mice were sacrificed and various organs and tumor were aseptically removed. Tissue samples were fixed in 4% paraformaldehyde and embedded in paraffin blocks and cut into thick slices. Finally, the slices were stained by hematoxylin/eosin (H&E) and observed under a microscope from Nikon Instruments (Tokyo, Japan [40]).

### Systemic toxicity analysis

To investigate changes in liver and kidney functions after P/LNPs-siRNA-MF and P/LNPs-siRNA–BM treatment, levels of AST, ALT, BUN and CRE in serum were determined. Briefly, venous blood was collected from treated mice and centrifuged at 10, 000 rpm for 10 min at 24 h after P/LNPs containing 2.5 mg siRNA per kg or saline treatment. Plasma samples were analyzed using a kit from NanJing JianCheng Bioengineering Institute (Nanjing, China) according to manufacturer’s instructions.

### Statistical analysis

Data were expressed as the mean ± S.D of triplicates unless otherwise indicated. Statistical significance was determined by Student’s *t* test. Significance levels are indicated as ^*^p < 0.05, ^**^p < 0.01 and ^***^p < 0.001 as compared with the corresponding control values.

## CONCLUSIONS

MF was used to produce P/LNPs with smaller size, narrower size distribution, lower positive zeta potential, which could more efficiently deliver siRNA into the tumor cells. P/LNPs made in MF method exhibited higher cellular uptake, greater inhibition of VEGF expression and greater tumor cell cytotoxicity compared with those produced by the conventional BM method. *In vivo*, the results revealed that P/LNPs-siRNA-MF displayed ideal blood circulating time, satisfactory antitumor activity, elevated *in vivo* gene silencing without causing systemic toxicity. Therefore, we believe P/LNPs-siRNA-MF may be a useful siRNA delivery vehicle for therapy of liver cancer.
